# Addressing diversity and complexity in the community engagement literature: The rationale for a realist review

**DOI:** 10.12688/wellcomeopenres.15525.2

**Published:** 2021-03-29

**Authors:** Emma Z.L. Richardson, Sunita V.S. Bandewar, Renaud F. Boulanger, Rukshan Mehta, Tinya Lin, Robin Vincent, Sassy Molyneux, Arisa Goldstone, James V. Lavery

**Affiliations:** 1 Centre for Ethical, Social and Cultural Risk, Li Ka Shing Knowledge Institute, Unity Health, 250 Yonge Street, Suite 600, Toronto, Ontario, M5B 2L7, Canada; 2 Health Research Methods, Evidence and Impact, McMaster University, Hamilton, Ontario, L8S 4K1, Canada; 3 Applied Health Research Centre, Li Ka Shing Knowledge Institute, Unity Health, 250 Yonge Street, Suite 600, Toronto, Ontario, M5B 2L7, Canada; 4 Health, Ethics and Law Institute, 422 Veer Savarkar Marg, Prabhadevi, Mumbai, 400 025, India; 5 Vidhyak Trust, C-5, Mantri Avenue, Pune, MH, 411 008, India; 6 Centre for Applied Ethics, McGill University, 2155 Guy Street, 223.08, Montréal, H3H 2R9, Canada; 7 Nutrition and Health Sciences PhD Program, Rollins School of Public Health and Laney Graduate School, Emory University, 1518 Clifton Road NE, CNR, Atlanta, GA, 30322, USA; 8 Department of Obstetrics and Gynecology, University of Calgary, Foothills Medical Centre, 1403 29th Street NW, Calgary, Alberta, T2N 2T9, Canada; 9 Independent researcher, Sheffield, UK; 10 REAL, Centre for Tropical Medicine and Global Health, Nuffield Department of Medicine, University of Oxford, Oxford, OS1 3SY, UK; 11 Department of Health Systems and Research Ethics, KEMRI/Wellcome Trust Research Programme, Kilifi, Kenya; 12 The Ethox Centre, Department of Public Health,, Oxford University, Old Road Campus, Headington, Oxford, OX3 7LF, UK; 13 The Centre for Tropical Medicine and Global Health, Nuffield Department of Medicine, University of Oxford, Old Road Campus, Headington, Oxford, OX3 7LF, UK; 14 Hubert Department of Global Health, Rollins School of Public Health & Center for Ethics, Emory University, 1518 Clifton Road NE, CNR 5007, Atlanta, GA, 30322, USA

**Keywords:** Community engagement, health research, low- and middle-income countries, realist review

## Abstract

In this research note we reflect on our failed attempt to synthesize the community engagement literature through a standard systematic review and explain our rationale for now embarking on a realist synthesis of community engagement in global health research. We believe this paper will be helpful for many who grapple with the lack of clarity about community engagement’s core elements and mechanisms.

## Introduction

Community engagement (CE) is increasingly recognized as an integral aspect of global health and global development research, building on early efforts by non-governmental organizations and community-based organizations to enhance the impact of their work through participatory and collaborative methods ^1– 6^. Support for CE activities in biomedical research budgets began in 1990 when the National Institute of Allergy and Infectious Diseases (NIAID) began to fund Community Advisory Boards (CAB) for its HIV prevention trials ^7^. The Bill & Melinda Gates Foundation and the Wellcome Trust have also supported CE strategies for their investments and research on CE. Most recently, the World Health Organization (WHO), following the 2015 Ebola outbreak, has formally incorporated community engagement into its International Health Regulations ^8, 9^.

Many major research initiatives now include community engagement activities, but clarity about the goals of CE and the understanding of how to achieve them remains underdeveloped ^10– 14^. This may be due, in part, to the fact that many early efforts at CE in global health and development challenged dominant trends by trying to privilege the perspective of their host communities ^6^. At the same time, the interest in community engagement draws unevenly on diverse histories of theory and practice in health and development, importing many, often unacknowledged, assumptions and distinctions about the aims and methods of engagement that have evolved in a range of different settings. As CE continues to gain standing in the eyes of funders and researchers in global health and global development, there is increasing urgency to clarify its core elements and the mechanisms through which it produces the relevant ethical and practical outcomes.

An obvious step in this direction is some form of systematic review of the CE literature. In this research note, we describe a realist synthesis that we are undertaking to inform our understanding of CE. First, we share our experiences with an unpublished literature review, which contributed to our conviction of the need for a realist review. We then detail some of the challenges and potential benefits of a realist synthesis and our current work. 

Between 2008–2009, we attempted to conduct a traditional systematic review of the community engagement literature. We had embarked on a series of case studies related to CE in global health and global development that aimed to generate rich descriptions of CE strategies in various research settings and to provide insights about what makes CE effective. At the time, we had already noted the lack of clarity in the literature, both about what CE is and how to conceptualize effectiveness for CE strategies ^10^. To inform the design of our case studies, and to look for any relevant insights from the literature about these fundamental questions, we set out to undertake a standard systematic literature review. The review was organized around three broad questions: (1) How is community engagement discussed in the relevant peer-reviewed academic literature? (i.e., what are the governing ideas and concepts?) (2) What CE activities/practices are described and/or suggested in the selected articles? (i.e., what *is* CE?) (3) How is the evaluation of community engagement approached/discussed? (i.e., what makes CE effective?)

## Methods

We began our review by identifying a string of relevant search terms through literature we were aware of, supplemented by a Scholars Portal thesaurus search, to ensure proper matching with database terms. Our initial string of terms related to CE was as follows: ‘Action Research’; ‘Citizen Participation’; ‘Collaborative Partnership’; ‘Collaborative Research Partnership’; ‘Community Action’; ‘Community Advisory Board’; ‘Community Advisory Committee’; ‘Community Consultation’; ‘Community Development’; ‘Community Engagement’; ‘Community Involvement’; ‘Community Liaison’; ‘Community Liaison Person’; ‘Community Participation’; ‘Community Research Support Groups’; ‘Community-Based Participatory Research’; ‘Community-Based Research’; ‘Exchange Theory’; ‘Participatory Research’; ‘Public Engagement’. In this first string, we did not include a similar string of terms based on the term ‘stakeholder’, which we intended to run separately. For each of these search terms, we conducted separate search runs in combination with key terms to explore the evaluation of CE. For example: ‘Community Engagement’ AND (Assessment OR Effectiveness OR Effectiveness evaluation OR Evaluation OR Process evaluation OR Relevance).

After assembling the search term structures, we conducted our preliminary searches in two phases. In the first phase, we ran these search structures through four databases: Scholars Portal, Scopus, Cochrane Review and Web of Knowledge. In the second phase, we ran the searches through three additional databases: International Bibliography of Social Science (IBSS), Anthropology Source and AnthroPlus. In these two phases we ran 147 separate searches. The aim in these preliminary searches was to get some sense of the scope of the potentially relevant literature. In total, the combined searches, after removing duplicates in our reference database, identified 98,618 individual papers.

Given the extraordinary scope of the literature, we abandoned any attempt to conduct and publish a traditional systematic review, but we persisted with a more selective approach to improve our theoretical sensitivity for the case studies we had begun. We first developed a strategy to screen the results for each search string individually. If a search string returned more than 150 manuscripts, we first used the RefWorks database tools to organize these chronologically from most recent to earliest. Then, the titles and, when necessary, the abstracts and the body of the 50 most recent articles were screened for relevance. When an article seemed to address our research questions, its bibliographical information was added to an online reference manager (RefWorks). The same steps were then followed using a second automated tool of the databases—relevance sorting—which, according to Scopus, means “sorting the results according to best match for your search terms.” This meant that for strings returning more than 150 articles, the 50 most recent publications and the 50 publications deemed by the databases to best match our search terms were screened. If a search string returned fewer than 150 results, all the articles were screened to increase coverage. After this preliminary screening, the number of manuscripts was reduced to 4,349. Because we were most interested in what it means to claim that CE is ‘effective’, we decided to adopt a pragmatic approach to distill the search results further. First, we adopted an explicit framing for papers that we considered to be of most immediate relevance to our work. We asked whether any paper was an “empirical study presenting primary data on the effectiveness of CE activities in the context of research”. Our rationale was that empirical studies would be easy to identify and that even if there were a large number of papers, it would be relatively easy to examine any reports that related to claims of effectiveness. RB further reduced the number by manually screening all article titles and, where applicable, abstracts. The body of the article was skimmed if relevance could not be determined by title and abstract alone. Through this process we reduced the number of papers to 153 ^15^. A second round of screening, which involved looking more closely at the body of the articles for some evidence of content reflected in our screening framework, further reduced the number of papers for full review to 49.

We subjected this sample of papers to detailed review, applying a review framework that we had developed prior to the execution of the initial searches. We selected the first five papers in this final sample and conducted a pilot review. Four reviewers (RB, SB, RM and JVL) used the draft framework to review these five papers, identified issues and problems associated with the framework, and deliberated about appropriate revisions. Disagreements were resolved by JVL. All papers reviewed in this pilot round were reviewed again with the revised framework, which consisted of 18 questions ( Table 1).

**Table 1.** Framework for systematic review.

Framework for Systematic Review How is ‘effectiveness’ of community engagement conceptualized & what are some of the good practices that should be used to foster effective engagement?	
Paper’s Bibliography (Author, Year, Title, Journal, Volume, Issue, Page Number)	
**1. Study site (country)**	
**2. Scope**	a. Local b. Regional c. National
**3. Research field**	a. Health b. Education c. Agriculture d. Forestry e. Other_________________________
**4. Type of paper**	a. Empirical (primary data) b. Non-empirical/Conceptual (no primary data) c. Literature Review (secondary analysis of primary sources)
**5. If the paper is based on primary data collection or secondary** **analysis, does it describe:**	a. A planned engagement intervention, with no evaluation b. A planned engagement intervention, with a structured evaluation (using clear indicators, either prospective or retrospective) c. A planned engagement intervention, with an unstructured evaluation (not using clear indicators, either prospective or retrospective) d. A non-planned engagement intervention, with no evaluation e. A non-planned engagement intervention, with a non-planned evaluation
**6. From whose perspective does the paper discusses community** **engagement?**	1. Community 2. Researchers 3. Both
**7. Does the paper offer a definition of ‘community’?**	Yes or No?
**8. If a definition of ‘community’ is offered, what is this definition?**	
**9. Does the paper state any specific aims for using community** **engagement?**	Yes or No?
**10. If the paper states any specific aims for using community** **engagement, what are they?**	
**11. Does the paper offer a definition of ‘community** **engagement’ or of what is considered community engagement?**	Yes or No?
**12. If a definition of ‘community engagement’ is provided,** **what is this definition – or what is considered community** **engagement?**	
**13. Does the paper define what ‘effectiveness’ of community** **engagement mean?**	Yes or No?
**14. If the paper defines ‘effectiveness’, what is the definition used?** **If the word ‘effectiveness’ is not used, indicate what related** **term (e.g.: success) was used.**	
**15. Does the paper describe ways to evaluate ‘effectiveness’ of** **community engagement?**	Yes or No?
**16. If ways to evaluate community engagement and effectiveness** **were described or suggested, what were they?**	
**17. Does the paper recommend specific community engagement** **activities or practices?**	Yes or No?
**18. If activities or practices are recommended, list them and** **identify if they are:** **a. Based on empirical findings (EF)** **b. Not based on empirical findings or described as** **‘self-evident’ (SE)**	
**19. List other relevant key points**	

## Results

One surprising insight from this exercise was how infrequently, and how poorly, the aims of CE are reported in individual studies. This finding provided an important framing for the project and helped us understand how little traction we were able to achieve around our three research questions.

*(1) How is community engagement discussed in the relevant peer-reviewed academic literature? (i.e., what are the governing ideas and concepts?)*

We drew three main conclusions about how CE was discussed in the sample of studies we identified. First, CE, in some form or other, is represented in an almost endless range of human endeavors, from politics to industrial relations to global finance to clinical drug trials. Because each domain and context of application has its own—usually implicit—assumptions about the proper goals of CE and conventions about language and appropriate processes, it makes aggregation of these experiences and comparative analyses extremely complicated. Second, there is no single academic or scholarly tradition that has an exclusive claim on CE. The selected 49 papers represented diverse fields and disciplines including: public health, HIV/AIDS, public administration and finance, drugs, indigenous people, forestry, urban planning, youth, ageing, disability, education, energy, occupational therapy, community development, military, development, and mixed disciplines. Reflecting this disciplinary diversity, there was significant variation in terminology, conceptualization, and framing of CE elements and goals, to the extent these were articulated. Third, the pervasiveness of CE and the many ways it is invoked and studied in the academic literature has given rise to an overwhelming volume of potentially relevant academic literature.

Compounding the variability in terminology, there were few efforts to clarify seemingly relevant differences, e.g., between ‘community engagement’, ‘community mobilization’, ‘community consultation’, among many others. The challenge was further complicated by the heavy reliance on broad, elastic concepts, which are amenable to multiple interpretations in the absence of some stipulation or specification. With a few notable exceptions, we were struck, as well, by the level of ‘taken-for-grantedness’ that permeated the initial batches of literature we retrieved in preparation for our more comprehensive searches. We found very few examples of careful explanations and elaborations of the relevant concepts, or circumspection about how the use of vague or ambiguous terminology might thwart efforts to draw reliable inferences, and these were exacerbated by the obviously complex interplay between concepts like ‘community’ and the highly varied contexts in which the studies and conceptual analyses were situated.

Although 33/49 articles made some attempt to describe the study’s relevant community, only nine did so explicitly. In most cases, the ‘community’ appeared to comprise the research participants and their immediate geography and the social affiliations that are the source of their shared identity, a common conceptualization of community in research ^16^. Whose perspective was applied to define the relevant community was not addressed explicitly in any of the selected articles.

*(2) What CE activities/practices are described and/or suggested in the selected articles? (i.e., what is CE?)*

Ironically, given the wide variability of concepts invoked about CE, we found a fairly narrow view of what activities and practices CE might encompass. Some of the leading activities we saw described as CE included: different forms of meeting; vague notions of participation, usually without much elaboration; or allusion to dialogue and deliberation with very little description of specific activities. There was a lack of clarity about how different concepts, such as ‘community mobilization’ and ‘community consultation,’ differ in practical terms, or whether they were, effectively, interchangeable.

The situation was complicated further by the aspirational nature of many of the descriptions, which obscured a clear, empirical view of the actual CE practices being reported. For example:

“True participation involves a process of personal as well as social transformation in which decision making takes place in the hand of the consumer group and social conditions are thereby affected or changed” (Boyce and Lysakc (2000) as summarized in Taylor, Braveman, & Hammel, 2004, p. 73) ^17^.
  “…a public participation process should attract a wide range of people to the process, allow participants to influence decisions and ensure that the entire process is open and that decisions from the process are transparent. Additionally, participants of the process should have clear instructions about their role and should have the resources and information available to assist with informed decision-making” ^18^.
*(3) How is the evaluation of community engagement approached/discussed? (i.e., what makes CE effective?)*

The ‘success’ or ‘effectiveness’ of CE was discussed, in varying degrees of explicitness, in four general ways: (1) in terms of the successful achievement of project goals and targets; (2) in terms of some forms of productive change or improvement in the host community; (3) in terms of general community/participant satisfaction captured in various qualitative, quantitative, or mixed methodologies; and (4) in terms of the formation of positive relationships in the host community. Given the lack of conceptualization of ‘community’ and ‘engagement’ in the articles, and the virtually infinite number of ways that the possible modes of engagement might affect change at individual and collective levels, the potential scope of CE’s impact seems significantly under-examined in our sample.

The majority of articles made either explicit or implicit reference to normative concepts, such as trust or transparency, in their findings or discussions. For example, Hunt (2007) suggests a possible summary measure of CE effectiveness, without stating so explicitly, “Trust should be viewed as a long-term goal of the decision-making process. Opportunities for shared learning may represent one method to assist in developing trust…” (p. 112).

Some authors also cautioned that what constitutes ‘effectiveness’ may vary from one context to another. For example, Charnley (2005) ^19^ notes that “effective community involvement techniques in one community may not work in another community” (p. 178).

Several authors lamented the lack of systematic evaluation relating to CE effectiveness:

“This brings us to the final conclusion of this paper – the need for situated studies of participatory appraisal experts and the analytic-deliberative space that they shape.” …. “Only through such critical inquiry and reflection will we have a chance of making public engagement in science that is truly democratic, fair, competent, and engenders wider social learning” ^20^.
  “Despite the encouraging evidence on citizen empowerment uncovered by this research, the evaluation and measurement of the effectiveness of citizenship activities remains an important area for systematic empirical investigation” ^21^.

## Discussion and conclusions

First, we expected divergence in terminology in our sample, since we had explicitly searched a wide range of search terms. However, our review reinforced our initial sense that these terms are often used interchangeably, or without sufficient stipulation of their meaning in the specific circumstances or contexts of application. Second, we encountered few explicit statements about the purpose of CE strategies described in the papers, and the terminology invoked about ‘engagement’ rarely corresponded to clearly differentiated processes or outcomes. Third, although the papers often recognized that the context of CE can influence the goals, processes and outcomes that are thought to be relevant, there was very little elaboration about what features of context are important or how they function as determinants of relevant or appropriate CE. Fourth, some papers recognized that what counts as ‘effective’ CE would vary according to the perspective of the observer. Yet there were few elaborations of differing perspectives or how, or why, these differences exist. Fifth, although many of the final sample of studies provided some definition or account of ‘community’, often in terms of pre-existing geographic or social associations, there were very few attempts to articulate the intended meaning of engagement, or mobilization, or partnership, or any of the other descriptors of the nature of engagement. And sixth, we believe that these observations help to explain why CE has been so resistant to conventional approaches to evaluation, to date.

Our failed attempt to synthesize the CE literature revealed the limits of approaches that rely heavily on the stability and reliable interpretation of key terms and concepts for areas such as CE, which are almost infinitely diverse and complex. This experience, and our decades-long involvement in a wide range of community engagement studies ^10– 14, 22^, has convinced us of the relative merits of a realist approach to a literature review. We therefore lay out, in the following sub-sections, the rationale for pursuing a realist review in this area.

## Rationale for a realist review

Community engagement is a complex, multi-stakeholder process that varies widely according to the nature and specific goals of the research, and with the unique constraints of the contexts of application. Realist synthesis is increasingly recognized as an effective process for consolidating evidence and learning from complex social processes and interventions, with notable successes in public health and community development ^23– 25^. Realist reviews identify and refine program theories, understandings of the role of important aspects of context—both of which are critically under-developed in CE scholarship—and how these relate to patterns of outcomes observed in the literature and in practice. The method begins with the premise that the same intervention may perform differently in different contexts and aims to elucidate “what works for whom in what circumstances” ^26^.

### Tackling complexity

Community engagement with health research is a complex multi-stakeholder process that is intimately affected by the social and cultural settings within which it takes place, including the history and perception of previous relationships and engagement. It is affected by the relationships that give rise to the funding and design of research programs, and by the political economy of relatively wealthy and powerful research institutions operating in contexts of poverty and underdeveloped health infrastructure. This is a quintessentially complex relational setting in which interacting and shifting social dynamics are the norm. 

Health and development practitioners have increasingly drawn on concepts and understandings from complexity science to move beyond traditional linear ‘command and control planning’ ^27, 28^ to recognize the role of emergence and feedback in social interactions, and thus the inadequacy of traditional linear and variable based quantitative analysis ^29, 30^. However, complexity concepts have been borrowed unevenly and inconsistently ^31^. Where some have responded to recognition of emergence by stressing that social interactions are radically contingent ^32, 33^, critical realists have argued that social interaction is not entirely contingent, but structured by identifiable social mechanisms, so ‘complexity sensitive’ or ‘complexity congruent’ approaches still hold the promise of cumulative learning and science ^34, 35^. Where the above is most explicitly argued in Archer (1995) ^36^.

Pawson (2013) ^37^ also makes a case for a ‘science’ of evaluation, arguing that complexity can be attended to analytically by using explicit program theory to help construct boundaries around any particular inquiry (rather than attempting to look at the whole system at once) and systematic attention to context, to help focus the gathering of evidence ^34^. One of us (JVL) has similarly defined CE as a ‘wicked problem’ and called for an implementation science for research ethics to improve our understanding of how research ethics strategies work ^11^. The same realist logic of inquiry can apply both to individual evaluations and to systematic reviews of many, providing a way to navigate the complexity and build cumulative learning, whilst also developing an understanding of the influences of context and the variation in outcomes across settings ^34^.

### Relevance of critical realism and the importance of context

Those grounding their work in critical realist and scientific realist schools of thought share a common recognition that social programs and interventions are “complex systems thrust amongst complex systems” (Pawson, 2006a, p. 35). The context within which social programs are carried out is seen as intimately tied up with how participants respond, and which mechanisms are triggered by an intervention. There is recognition that no intervention is the ‘same’ twice, and that context will be part of what influences the pattern of outcomes seen across settings. Context has an ‘active’ role in determining which configuration of mechanisms unfold in response to an intervention, so that context is not something that needs to be screened out as a ‘confounding’ factor in any review, but rather something that needs to be better understood.

For this reason, realist synthesis takes context seriously, and seeks to gather a systematic picture of all the contingencies affecting how projects and programs play out. It does this by beginning with an explicit program theory or theories and by gathering evidence around these theories to adjudicate among them. In this way, the program theory underpinning the intervention can be iteratively refined. Rather than definitive verdicts of success or failure, a realist evaluation approach looks for configurations of context, mechanism and outcomes to build a fuller picture of the varying fortunes of any particular intervention across time and place. For our realist review, we have decided to initially search literature in one field only: Malaria. This will allow us to narrow the number of fields we are considering simultaneously, at least initially. Focusing on this one field in public health will also allow us to focus the sources of working definitions for Community Engagement at the outset.

### Avoiding the hierarchy of evidence

The focus on program theory, and the recognition that there may be ‘portable program theories’ across a range of intervention and implementation contexts provides a way to navigate both the complexities of practice and the literature of CE. By taking program theory and the “underpinning mechanism of action” as the primary unit of analysis, realist synthesis “maximizes learning across policy, disciplinary and organizational boundaries” and allows learning from broader bodies of literature ^26^. At the same time, realist synthesis shifts the focus away from the traditional hierarchy of evidence underpinning systematic reviews. Critical realism stresses the configurational nature of causality in social practice (context-mechanism-outcome) and the value of qualitative methods for directly observing these causal processes. It also stresses the advantages of this approach over variable-focused methods, which typically rely on indirect inference about causal processes from measured differences in variables over the course of an intervention ^38^. The realist recognition of the role of context also means that the tendency for traditional systematic review to average out differences in context is deliberately avoided. Realist review also draws on a greater range of evidence, including insights from grey literature and the knowledge of practitioners and those involved in projects on the ground. This does not necessarily reduce the challenges of doing a systematic review, and means that transparency and documentation in the process of the review is vital ^39^.

The protocol for our realist synthesis of the community engagement literature has been published, with the methodological details described therein ^40^.

As we continue with this realist synthesis, we are reflexive about why we believe this is the right method to tackle the many dimensions of complexity in systematizing the CE literature. We hope this reflection will hearten others who are similarly frustrated by the lack of coherence in the CE literature but equally optimistic about the promise community engagement holds for sustainability and effectiveness in global health research.

## Data availability

### Underlying data

DANS-EASY: Data for systematic review about effectiveness in community engagement. https://doi.org/10.17026/dans-zq9-25bm ^15^.

Data are available under the terms of the Creative Commons Zero “No rights reserved” data waiver (CC0 1.0 Public domain dedication).
