# Supplementary material for: Addressing diversity and complexity in the community engagement literature: The rationale for a realist review
Source: Wellcome Open Res. 2021 Mar 29;5:1. Originally published 2020 Jan 7. [Version 2] doi: 10.12688/wellcomeopenres.15525.2 (PMC8474101; doi:10.12688/wellcomeopenres.15525.2)
Supplement: Peer review history

# Peer review: Reviewer response for version 2

**DOI:** 10.21956/wellcomeopenres.17850.r43283

**Published:** 2021-09-23

**Authors:** Morenike Oluwatoyin Folayan

**Affiliations:** 1 Obafemi Awolowo University, Ile-Ife, Nigeria

The manuscript reads well.

Is the work clearly and accurately presented and does it cite the current literature?

Partly

If applicable, is the statistical analysis and its interpretation appropriate?

Not applicable

Are all the source data underlying the results available to ensure full reproducibility?

Yes

Is the study design appropriate and is the work technically sound?

Partly

Are the conclusions drawn adequately supported by the results?

Partly

Are sufficient details of methods and analysis provided to allow replication by others?

Partly

Reviewer Expertise:

Ethics of community engagement in research

I confirm that I have read this submission and believe that I have an appropriate level of expertise to confirm that it is of an acceptable scientific standard.

# Peer review: Reviewer response for version 2

**DOI:** 10.21956/wellcomeopenres.17850.r43282

**Published:** 2021-03-30

**Authors:** Nicola Desmond

**Affiliations:** 1 Liverpool School of Tropical Medicine, Liverpool, UK

The article has been revised following reviewer comments and is now excellent.

Is the work clearly and accurately presented and does it cite the current literature?

Yes

If applicable, is the statistical analysis and its interpretation appropriate?

Not applicable

Are all the source data underlying the results available to ensure full reproducibility?

No

Is the study design appropriate and is the work technically sound?

Yes

Are the conclusions drawn adequately supported by the results?

Yes

Are sufficient details of methods and analysis provided to allow replication by others?

Yes

Reviewer Expertise:

medical anthropology, risk, uncertainty, community engagement, technologies, qualitative methods, acceptability, treatment seeking, behaviour change, process and impact evaluation

I confirm that I have read this submission and believe that I have an appropriate level of expertise to confirm that it is of an acceptable scientific standard.

# Peer review: Reviewer response for version 1

**DOI:** 10.21956/wellcomeopenres.16994.r37497

**Published:** 2020-01-09

**Authors:** Nicola Desmond

**Affiliations:** 1 Liverpool School of Tropical Medicine, Liverpool, UK

This is an interesting and honest account of a failed attempt to conduct a standard systematic review of the literature on community engagement. The paper states explicitly and openly that it is an account of failure as the first half of a rationale for conducting a realist synthesis of community engagement literature. The second half of the rationale comprises an account of complexity and context in engagement (and other) programmes that clearly indicates the need (as described) for a more nuanced critical realist approach to community engagement synthesis. This is an original piece of work, refreshingly honest about the difficulties inherent in conducting and reporting community engagement work including for example challenges in definitions. Given my own work around community engagement in the context of a large medical research institution in a very low resource setting and the constant challenges identified both in engagement as a practical issue and as a political issue, the paper may help in formulating a stronger and more convincing response to 'standard' ascription to a particular approach and the assumption that replication (across studies and settings (e.g. urban/rural) is necessarily going to be a success.

 Given all of this the paper was a pleasure to read. My only (slight) criticism is that the 'findings' of the initial (failed) synthesis are summarised rather than presented holistically - I would have been interested in seeing the list of 49 studies that made it through the screening process - perhaps these could be included as an appendix to the paper, available online.

 Otherwise I believe this submission provides a useful and original addition to the increasing body of literature recognising the difficulties inherent in community engagement concepts, practice and evaluation and provides a possible contribution to improving work and understanding (and ultimately ethics of practice) in this area of community engagement.

Is the work clearly and accurately presented and does it cite the current literature?

Yes

If applicable, is the statistical analysis and its interpretation appropriate?

Not applicable

Are all the source data underlying the results available to ensure full reproducibility?

No

Is the study design appropriate and is the work technically sound?

Yes

Are the conclusions drawn adequately supported by the results?

Yes

Are sufficient details of methods and analysis provided to allow replication by others?

Yes

Reviewer Expertise:

medical anthropology, risk, uncertainty, community engagement, technologies, qualitative methods, acceptability, treatment seeking, behaviour change, process and impact evaluation

I confirm that I have read this submission and believe that I have an appropriate level of expertise to confirm that it is of an acceptable scientific standard.

# Peer review: Reviewer response for version 1

**DOI:** 10.21956/wellcomeopenres.16994.r37496

**Published:** 2020-01-07

**Authors:** Morenike Oluwatoyin Folayan

**Affiliations:** 1 Obafemi Awolowo University, Ile-Ife, Nigeria

The authors have indeed tried to address a critical concept in the field of research: a concept that should inform researcher and participants/participants’ community interactions. I am not surprised (and I feel certain the authors were not surprised with the outcome of their finding for a number of reasons:

1. The phrase ‘Community engagement’ has not evolved into a concept. It is still used as a literary phase. In the absence of concrete efforts at evolving a concept that becomes nominally used in the research field, the findings as identified in the literature is not surprising. There has been significant efforts to try and nominalize the concept ‘stakeholder engagement’, which the authors will likely assume is synonymous with the phrase ‘community engagement’ in the biomedical HIV prevention field. Significant efforts have been made over the years. Even as at yet, the concept ‘Stakeholder engagement’ is still evolving.
2. Even with normalized phrases, the meanings can differ across field. The nominalized concept and its use in a field can differ somewhat in another field. The attempt made by the authors to study the conceptualization of community engagement across fields will therefore comes with the challenges they have experienced. I suggest they should focus on studying *How is ‘effectiveness’ of community engagement conceptualized & what are some of the good practices that should be used to foster effective engagement *in a specific field; and possibly compare research outcomes in a research field with its use in other fields of research
3. The authors also notes that terminologies were used *interchangeably*. I query their measure of *interchangeably *for a concept remains undefined. There was no evidence suggestive that specific phrases connotes specific meanings. The reflection on interchangeable use of terminology reflects a bias towards what specific terminologies should reflect.
4. The authors also did not share with the readers their conceptualization of community engagement. The research attempting to measure effectiveness of community engagement conceptualization imagines ‘community engagement’ is defined. I assume the ‘authors have a working concept of ‘community engagement’ against which the measure of effectiveness is drawn. I would have loved to see a reflection on some common threads in the use of the terminology across fields, or populations or specific spheres. This will help the field as it works its way through to nominalizing the concept ‘community engagement’.  

I like the discussion on tackling complexity and the relevance of context. However, I think the discussion only reinforces the need to first conceptualise *community engagement* and then recognize that its practice and the measure of its ‘effectiveness’ can differ by context. Reminds me of defining principles but the implementation of these principles are defined by context. Right or wrong then becomes context and situation specific. This does not rule out the need to conceptualise. In effect, *verdicts of success or failure *can be defined per context.

 I think McQueen and Day have both being doing a lot of work on measures for community engagement in biomedical HIV prevention research. This may align with the efforts by the authors to conceptualise ‘effectiveness of community engagement’. I suggest a review of the literatures my these authors can help shape their thoughts around this manuscript. The suggested framework for systematic review is faulty if the concept of what is being reviewed is unknown – cannot be defined. I argue that this concept is defined better in some field than others and thus traditional systematic reviews of community engagement concepts can be conducted in fields like biomedical HIV prevention research.

Is the work clearly and accurately presented and does it cite the current literature?

Partly

If applicable, is the statistical analysis and its interpretation appropriate?

Not applicable

Are all the source data underlying the results available to ensure full reproducibility?

Yes

Is the study design appropriate and is the work technically sound?

Partly

Are the conclusions drawn adequately supported by the results?

Partly

Are sufficient details of methods and analysis provided to allow replication by others?

Partly

Reviewer Expertise:

Ethics of community engagement in research

I confirm that I have read this submission and believe that I have an appropriate level of expertise to confirm that it is of an acceptable scientific standard, however I have significant reservations, as outlined above.

## References

[1] : Stakeholder engagement to inform HIV clinical trials: a systematic review of the evidence.*J Int AIDS Soc*.21 Suppl 7: 10.1002/jia2.25174e2517410.1002/jia2.2517430334358PMC6192899

[2] : Stakeholder engagement toolkit for HIV prevention trials. *FHI*.2012; Reference source

[3] : Strengthening stakeholder engagement through ethics review in biomedical HIV prevention trials: opportunities and complexities.*J Int AIDS Soc*.21 Suppl 7: 10.1002/jia2.25172e2517210.1002/jia2.2517230334604PMC6193317

[4] : Evaluating community engagement in global health research: the need for metrics.*BMC Med Ethics*.2015;16: 10.1186/s12910-015-0033-94410.1186/s12910-015-0033-926126899PMC4488111

# Response

**Published:** 2021-08-31

**Authors:** Emma Richardson

**Affiliations:** St. Michael's Hospital, Toronto, Canada

Responses from first author **in bold next to each point**:

 The authors have indeed tried to address a critical concept in the field of research: a concept that should inform researcher and participants/participants’ community interactions. I am not surprised (and I feel certain the authors were not surprised with the outcome of their finding for a number of reasons:

1. The phrase ‘Community engagement’ has not evolved into a concept. It is still used as a literary phase. In the absence of concrete efforts at evolving a concept that becomes nominally used in the research field, the findings as identified in the literature is not surprising. There has been significant efforts to try and nominalize the concept ‘stakeholder engagement’, which the authors will likely assume is synonymous with the phrase ‘community engagement’ in the biomedical HIV prevention field. Significant efforts have been made over the years. Even as at yet, the concept ‘Stakeholder engagement’ is still evolving. **Agreed.  This was the motivation for trying to synthesize the literature with a systematic review.**
2. Even with normalized phrases, the meanings can differ across field. The nominalized concept and its use in a field can differ somewhat in another field. The attempt made by the authors to study the conceptualization of community engagement across fields will therefore comes with the challenges they have experienced. I suggest they should focus on studying *How is ‘effectiveness’ of community engagement conceptualized & what are some of the good practices that should be used to foster effective engagement *in a specific field; and possibly compare research outcomes in a research field with its use in other fields of research. **Thank you for this suggestion for how to narrow the study.  We decided to test whether a realist synthesis might be able to shed light on effectiveness in community engagement.  We indeed focused on one specific field in health research: malaria.**
3. The authors also notes that terminologies were used *interchangeably*. I query their measure of *interchangeably *for a concept remains undefined. There was no evidence suggestive that specific phrases connotes specific meanings. The reflection on interchangeable use of terminology reflects a bias towards what specific terminologies should reflect. **We gave examples for how we found these terms were used interchangeably.  Agreed the lack of standard definitions of community engagement, nor agreement on its aims, mechanisms and outcomes, complicates matters.**
4. The authors also did not share with the readers their conceptualization of community engagement. The research attempting to measure effectiveness of community engagement conceptualization imagines ‘community engagement’ is defined. I assume the ‘authors have a working concept of ‘community engagement’ against which the measure of effectiveness is drawn. I would have loved to see a reflection on some common threads in the use of the terminology across fields, or populations or specific spheres. This will help the field as it works its way through to nominalizing the concept ‘community engagement’. **To address one reviewer’s point about the definition of Community Engagement, we’ve typically used the Centres for Disease Control definition when pressed, but the point, perhaps, is that we were not convinced by past definitions, hence conducting this realist synthesis.**

I like the discussion on tackling complexity and the relevance of context. However, I think the discussion only reinforces the need to first conceptualise *community engagement* and then recognize that its practice and the measure of its ‘effectiveness’ can differ by context. Reminds me of defining principles but the implementation of these principles are defined by context. Right or wrong then becomes context and situation specific. This does not rule out the need to conceptualise. In effect, *verdicts of success or failure *can be defined per context. **Realist synthesis very much attempts to recognize and draw richness from context, while also emphasizing the importance of conceptualising - the difference is that the conceptualizing focuses at the level of mechanism causing the outcome in different contexts.**

 I think McQueen and Day have both being doing a lot of work on measures for community engagement in biomedical HIV prevention research. This may align with the efforts by the authors to conceptualise ‘effectiveness of community engagement’. I suggest a review of the literatures my these authors can help shape their thoughts around this manuscript. The suggested framework for systematic review is faulty if the concept of what is being reviewed is unknown – cannot be defined. I argue that this concept is defined better in some field than others and thus traditional systematic reviews of community engagement concepts can be conducted in fields like biomedical HIV prevention research. **This is a good point.  The original systematic review tried to understand the various definitions in play, but as you have identified community engagement is better defined in some fields than others.  For the subsequent realist review we narrowed to looking at malaria health research.**

**Thank you for your comments and suggested revisions.  We think the concerns you raise are ones we also identified in the original systematic review, and are what led us to consider the promise of a realist synthesis to instead focus on mechanisms of effective community engagement in malaria health research, with conceptual findings stemming from considering context-mechanism-outcome configurations.**
